# Regulating Multifunctional Oxygen Vacancies for Plasma‐Driven Air‐to‐Ammonia Conversion

**DOI:** 10.1002/anie.202508240

**Published:** 2025-05-12

**Authors:** Wanping Xu, Jiaqian Wang, Tianqi Zhang, Jungmi Hong, Qiang Song, Zhongkang Han, Patrick Cullen

**Affiliations:** ^1^ School of Chemical and Biomolecular Engineering The University of Sydney Sydney New South Wales 2006 Australia; ^2^ School of Material Science and Engineering Zhejiang University Hangzhou Zhejiang 310027 China

**Keywords:** Ammonia production, Electrochemistry, Nitrogen fixation, Oxygen vacancies, Plasma chemistry

## Abstract

Current ammonia (NH₃) synthesis is hindered by challenges including N₂ activation, NH₃ separation and process complexity. Here, we report a plasma‐electrochemical process for the production of gaseous ammonia from air generated NO*_x_*, decoupled from processes employing liquid phase intermediaries such as NO₃⁻ and final product (NH_4_^+^). Importantly, this process uses air for scalable ammonia production under ambient conditions and directly produces gaseous NH₃ which facilitates efficient product separation. For NO*_x_* reduction to NH₃, we propose a universal strategy combining plasma pretreatment and wet chemical calcination to introduce multifunctional oxygen vacancies. The resulting highly defective Fe₂O₃ nanoparticles on Cu achieves a significant ammonia production rate of 628 nmol·s⁻¹·cm⁻^2^, along with nearly 100% faradaic efficiency.

## Graphical abstract

High defective Fe_2_O_3_ nanoparticles on Cu synthesis by plasma introduced multifunctional oxygen vacancies achieving 100% NH_3_ selectivity through a plasma‐electrochemical system capable of directly reducing air to gaseous NH_3._

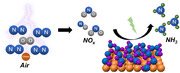

## Introduction

**Figure 1:**
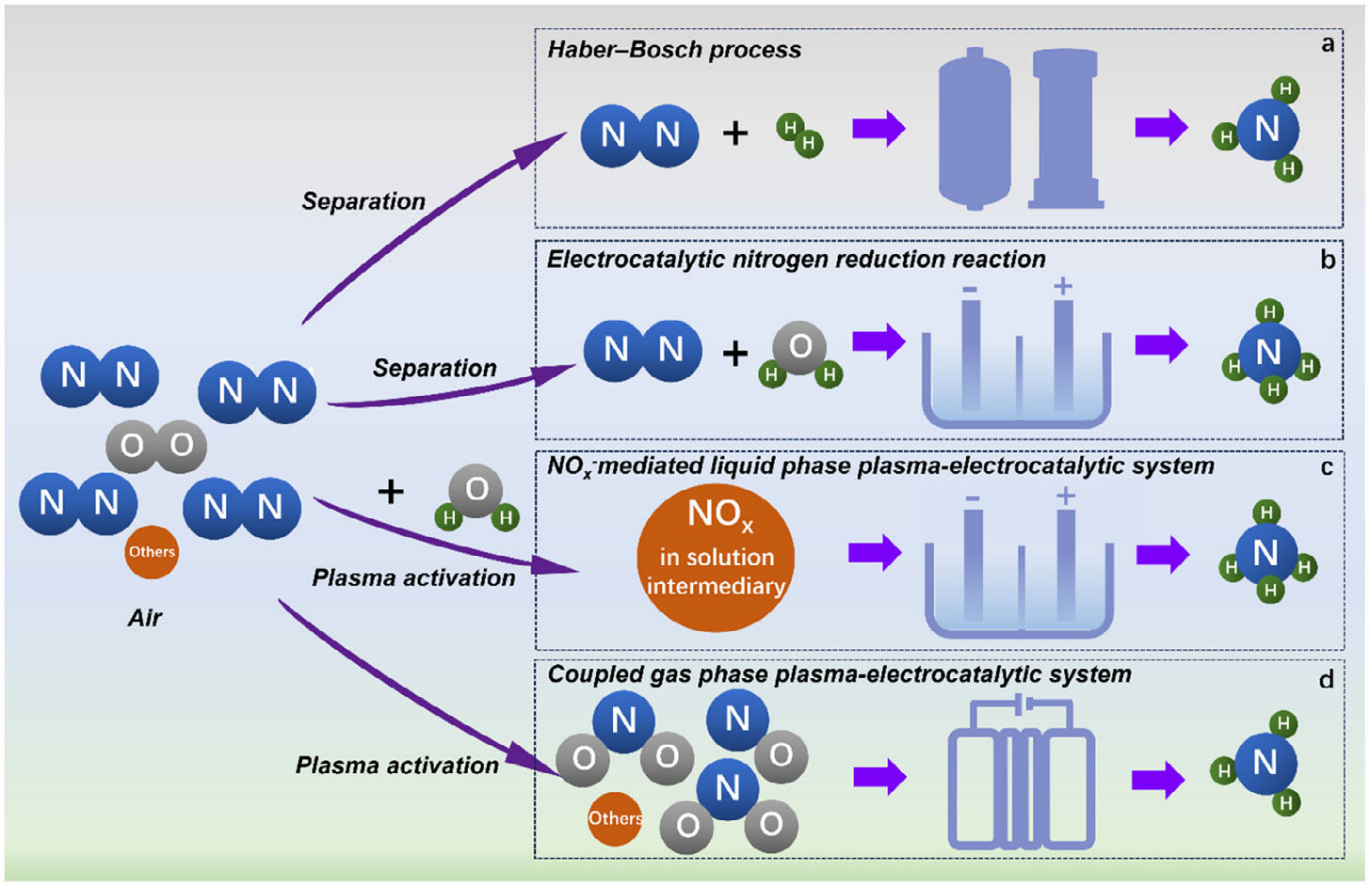
Four nitrogen fixation routes.

NH₃ is one of the most important chemicals globally, used as a fertiliser for approximately 50% of global food production and supporting 40% of the world's population.^[^ ^1^, ^2^ ^]^ Given that NH_3_ can be cracked to produce hydrogen and easily transported, it is also a technologically viable alternative fuel.^[^ ^3^, ^4^ ^]^ Ninety percent of global NH_3_ production relies on the Haber–Bosch (H‐B) method (Figure 1a), which operates at high temperatures and pressures using high‐purity N₂ and H₂ as raw materials.^[^ ^5^, ^6^ ^]^ The high energy consumption and CO₂ emissions derived from pure H₂ production, coupled with harsh reaction conditions, make it urgent to seek alternatives to the H‐B method.

Many attempts at the electrocatalytic nitrogen reduction reaction (eNRR) have been made to synthesise NH₃ under mild conditions using renewable electricity (Figure 1b). However, eNRR is still hampered by the intrinsic inertness and low solubility of N₂, as well as strong competing reactions in aqueous solutions.^[^ ^7^ ^]^ Nonthermal plasma (NTP) has been employed in recent eNRR processes as it can activate inert N₂ with low energy input.^[^ ^8^ ^]^ Considering NTP is more suited for oxidation reactions than chemical reduction and that nitrate (NO₃⁻) and nitrite (NO₂⁻) solubility in water is nearly 40 000 times that of N₂,^[^ ^9^, ^10^, ^11^ ^]^ N₂ and O₂ are used to produce NO*_x_* through the NTP process. After absorption and separation, NO*_x_* ⁻ in the electrolyte can be electrochemically converted to NH₃ in an integrated system (Figure 1c).^[^ ^12^, ^13^ ^]^ However, using N₂ and O₂ as precursors requires their separation from air, which increases raw material costs even for small‐scale ammonia production.^[^ ^14^ ^]^ Therefore, approaches where air is directly activated to produce NO*_x_* and the resultant NO*_x_* intermediaries reduced into NH₄⁺ via electrochemical conversion are attractive.^[^ ^10^, ^15^ ^]^

Although NTP‐induced NO*_x_* ⁻ in the electrolyte are considered as more reactive intermediates—promising better reaction kinetics for NH₃ synthesis due to their relatively low dissociation energy and enhanced mass transport in aqueous electrolytes^[^ ^14^ ^]^—when it comes to real‐world applications, there are still two issues to address: 1) NO₃⁻ and NO₂⁻ in the electrolyte or absorbent solution require post‐treatment to prevent environmental contamination, and 2) the produced NH₄⁺, as the water‐soluble form of NH₃, necessitates the extraction of parts per million amounts of NH₃ from the electrolyte after the reaction via regenerated resins or air stripping, which results in significant NH₃ loss.^[^ ^16^ ^]^ Considering the efficient separation and utilisation of NH₃ in the gas phase, we propose a rationally coupled gas‐phase plasma‐electrocatalytic (GP‐PE) system capable of direct air‐to‐NH₃ conversion (Figures 1d and S1b). The obtained gas‐phase NH₃ can be efficiently separated and captured using crystalline porous materials and porous organic polymers for further utilisation.^[^ ^17^ ^]^

Cu‐based catalysts have demonstrated significant potential among the various electrocatalysts tested for NO*_x_* RR due to copper's high tunability, low‐temperature activity and natural abundance.^[^ ^18^, ^19^, ^20^ ^]^ Additionally, Cu exhibits excellent redox properties due to the variable valence states of Cu^2^⁺ and Cu⁺, making it an ideal support for combining with other elements.^[^ ^21^ ^]^ Based on this, various strategies have been employed to further enhance the activity and selectivity of Cu‐based catalysts, including defect engineering,^[^ ^22^ ^]^ alloying,^[^ ^23^ ^]^ molecular decoration^[^ ^24^ ^]^ and doping.^[^ ^25^, ^26^ ^]^ Inspired by the use of Fe₂O₃‐based catalysts for selective catalytic reduction of NO*_x_* for the SCR process in the presence of NH₃,^[^ ^27^ ^]^ we developed highly defective Fe₂O₃ nanoparticles on Cu (denoted as Fe₂O₃ NPs/Cu) as an electrocatalyst. Following facile air activation by plasma, the Fe₂O₃ NPs/Cu catalyst achieved 100% faradaic efficiency (FE) in converting NO*_x_* to NH₃, reaching a significant NH₃ yield of 628 nmol·s⁻¹·cm⁻^2^. The origins of activity and structure–property relationships of the Fe₂O₃ NPs/Cu catalyst were investigated through a series of physical characterisations and theoretical calculations.

## Results and Discussion

### Formation Mechanism of Fe₂O₃ NPs/Cu Catalyst

**Figure 2:**
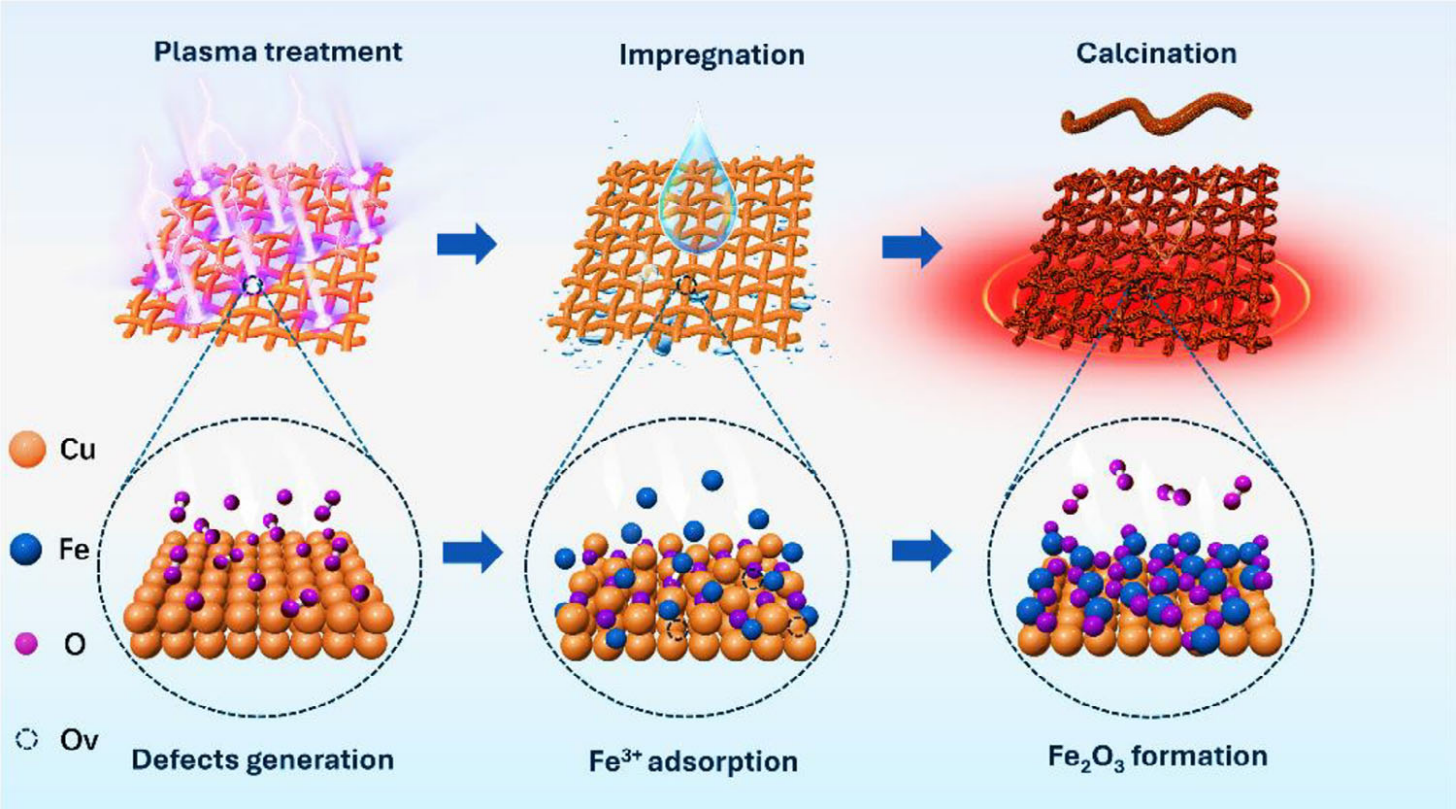
Schematic diagram showing the process of plasma treatment, wet chemical impregnation and calcination.

A highly defective Fe₂O₃ NPs/Cu catalyst was rationally prepared through a multifunctional oxygen vacancy‐modulated process, as illustrated in Figure 2. Initially, the cleaned pristine Cu (denoted as P‐Cu) was treated in an oxygen plasma atmosphere to introduce defects with the obtained sample denoted as Cu*_x_* O/Cu. In this process, oxygen molecules are excited by the high‐energy plasma, generating highly reactive species such as O⁻ ions, O atoms and O₃ molecules.^[^ ^28^ ^]^ These reactive oxygen species interact with Cu, leading to surface oxidation. During treatment, defects form as certain high‐energy oxygen species fail to fully bond with the Cu, resulting in the formation of oxygen vacancies (OVs) and unsaturated Cu sites. Through a further wet impregnation process, the OVs and under‐coordinated Cu atoms enhance surface chemical reactivity, providing increased adsorption capacity for Fe^3^⁺. Cu^2^⁺ in CuO, having a similar oxidation state to Fe^3^⁺ and serving as an active site that accepts electrons from Fe^3^⁺, interact with Fe^3^⁺ via electrostatic attraction. Stable ionic bond formation facilitates firm adsorption.^[^ ^29^ ^]^ Although the oxidation state of Cu⁺ in Cu₂O differs from Fe^3^⁺, Cu₂O can still indirectly enhance Fe^3^⁺ adsorption by modulating the surface's electron distribution. The coexistence of OVs and Cu⁺ helps to redistribute the surface charge, making it easier for Fe^3^⁺ to approach and adsorb onto Cu^2^⁺ sites. Consequently, the synthetic effects of OVs and Cu*_x_* O cause surface electronic structure redistribution, generating localised negative charges that facilitate Fe^3^⁺ adsorption and the formation of stable Fe–O–Cu bridge bonds on the surface.^[^ ^30^, ^31^ ^]^

In the final calcination process, the high surface energy associated with OVs allows these sites to serve as stabilising centres for adsorbates, making Fe atoms preferentially adsorb and nucleate at the OVs. The limited size of the OVs effectively regulates the local electronic structure, thereby inhibiting the growth of anchored particles and leading to the formation of small Fe₂O₃ nanoparticles.^[^ ^32^ ^]^ Additionally, under a nitrogen atmosphere, the low local oxygen concentration and suppressed surface reaction kinetics prevent further diffusion and aggregation of Fe₂O₃ particles, ensuring their uniform distribution across the Cu surface. Due to the limited Fe concentration, some OVs that do not fully participate in the reaction remain, and the reduction of Cu*_x_* O to metallic Cu generates additional OVs, collectively providing active sites for subsequent electrocatalytic reactions.

### Synthesis and Characterisation of Fe₂O₃ NPs/Cu

**Figure 3:**
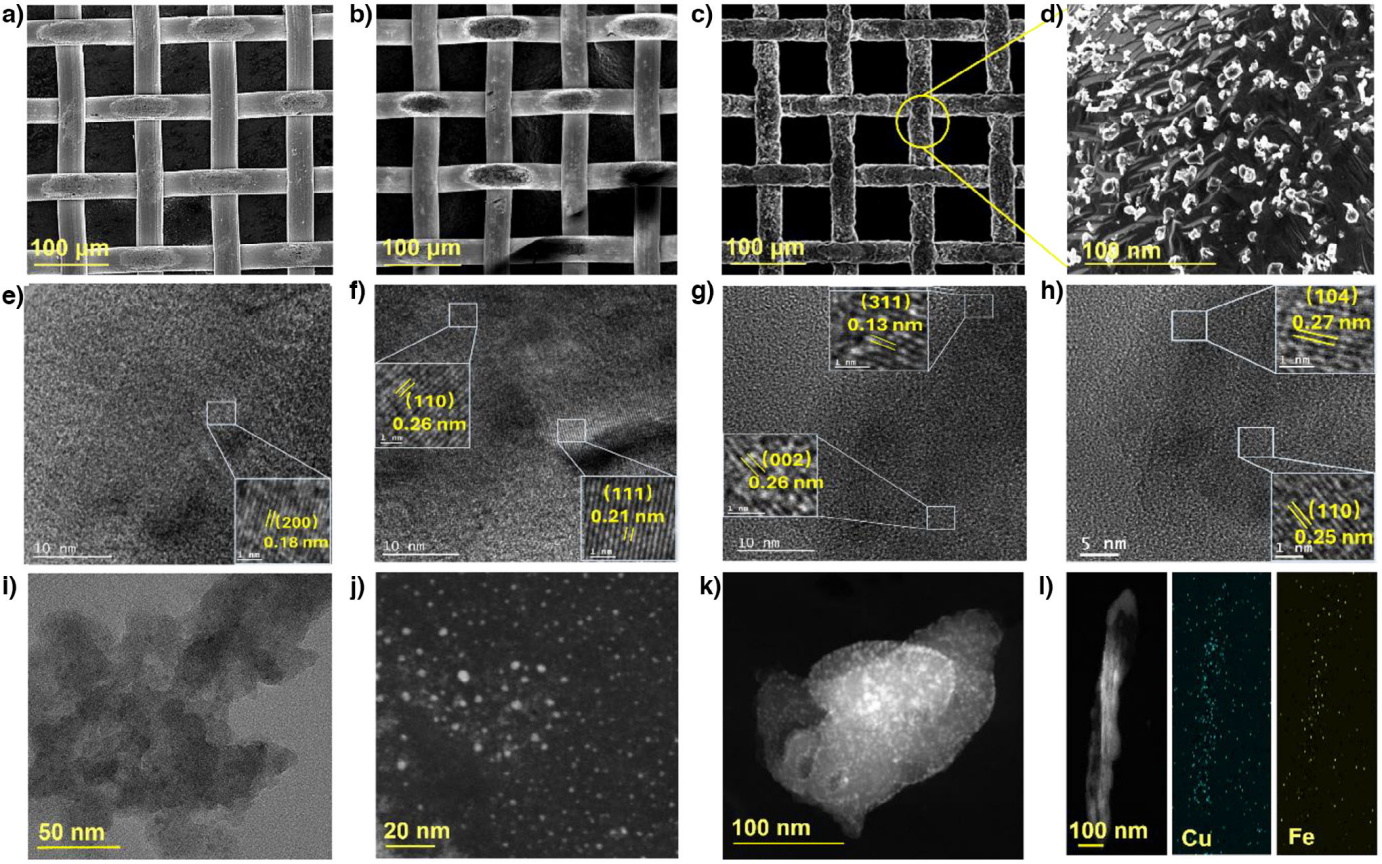
a) SEM images of P‐Cu, b) Cu*_x_* O/Cu, c) and d) Fe₂O₃ NPs/Cu. e) and f) HRTEM images of P‐Cu, g) Cu*_x_* O/Cu and h) Fe₂O₃ NPs/Cu. i) TEM image of Fe₂O₃ NPs/Cu. j)–l) STEM image and corresponding EDS mapping results of Fe₂O₃ NPs/Cu.

The samples were first morphologically characterised using scanning electron microscopy (SEM). Figures 3a and S2a (Supporting Information) show images of P‐Cu, whereas Figures 3b and S2b show that of Cu*_x_* O/Cu. The Cu*_x_* O/Cu surface is noticeably rougher than P‐Cu and this is thought to be caused by the effect of high energy ions and free radicals. The increased roughness provides more active sites for the subsequent adhesion of Fe^3^⁺. The images of Fe₂O₃ NPs/Cu in Figure 3c,d show that the surface of Cu is uniformly covered with nanoparticles after calcination. Figure S3 presents the corresponding energy‐dispersive X‐ray spectroscopy (EDS) of Cu, O and Fe. As seen from these figures, the surface oxygen concentration has significantly increased after plasma treatment and Fe is successfully loaded after calcination, confirming that plasma‐treated Cu is an ideal matrix providing sufficient anchoring sites for Fe. Figure S4 compares their X‐ray diffraction (XRD) patterns, in which P‐Cu exhibits the diffraction peaks characteristic of face‐centred cubic crystal structure Cu (PDF#04‐0836), whereas a subtle minor peak at 36.4° in Cu*_x_* O/Cu can be attributed to Cu₂O with (111) orientations.^[^ ^33^, ^34^ ^]^ This suggests that partial oxidation of Cu has occurred, with phase changes happening more on the surface than in the bulk material during the plasma treatment process. The absence of an obvious characteristic peak of Fe₂O₃ may be partly due to the low content and small size of the Fe₂O₃ nanoparticles. It should be emphasised that although no diffraction peaks appear, it does not necessarily imply the absence of Fe crystal forms, as it is challenging to confirm the crystal structure by XRD when the crystal size is small due to broad peaks and low signal‐to‐noise ratios.^[^ ^35^ ^]^

Thus, high‐resolution transmission electron microscopy (HRTEM) provides crucial evidence for identifying the oxide state. For P‐Cu, lattice spacings of 0.26 and 0.21 nm correspond to the (110) and (111) planes of Cu (Figure 3e,f), respectively.^[^ ^36^ ^]^ The grain boundary structures observed at 0.26 nm for CuO (002) and 0.13 nm for Cu₂O (311) suggest the existence of Cu^2^⁺/Cu⁺ interface structures (Figure 3g).^[^ ^37^ ^]^ These interface features, coupled with partial oxidation, are critical in promoting the formation of active sites and facilitating the generation of oxygen vacancies (OVs).^[^ ^38^ ^]^ The lattice fringe spacings of 0.25 and 0.27 nm correspond to the (110) and (104) crystallographic planes of the Fe₂O₃ crystal (Figure 3h).^[^ ^39^ ^]^ Transmission electron microscopy (TEM) and scanning transmission electron microscopy (STEM) images reveal that the small particles on Fe₂O₃ NPs/Cu are below 10 nm in size (Figure 3i,j). STEM images show bright dots, inferred to be Fe, as its atomic number is smaller than that of Cu (Figure 3k).^[^ ^40^ ^]^ This confirms that Fe₂O₃ primarily exists as well‐isolated nanoparticles with sizes smaller than 10 nm, although a small number of clusters may also be observed. Elemental analysis based on STEM image further verifies the uniform distribution of Fe₂O₃ particles or clusters across the entire Cu surface (Figure 3l). It should be noted that some areas of the Cu are exposed due to the detachment of nanoparticles during sonication in the sample preparation process.

**Figure 4:**
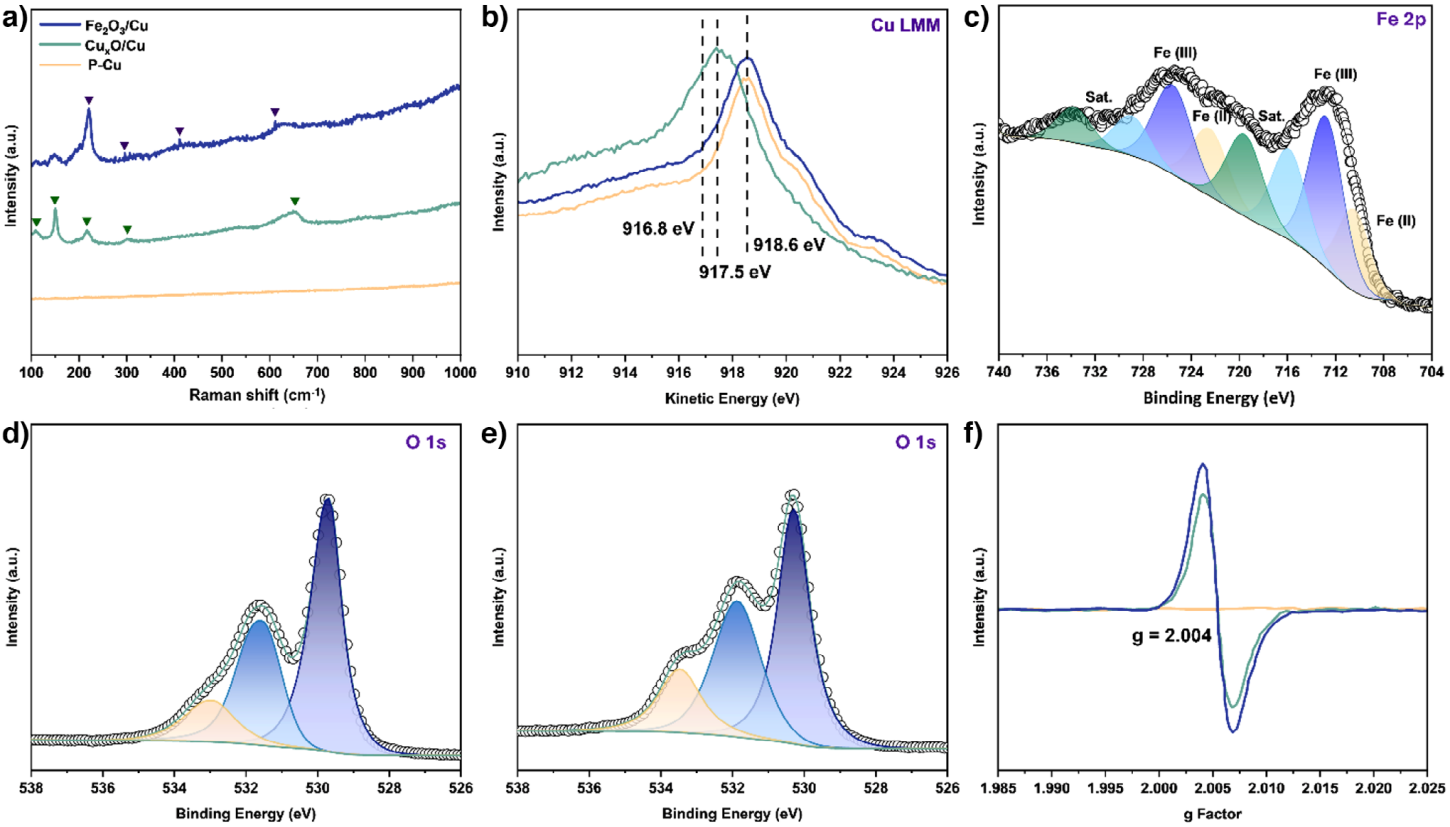
a) Raman spectra. b) Cu LMM XPS spectra of P‐Cu, Cu*_x_* O/Cu and Fe₂O₃ NPs/Cu. c) Fe 2p XPS spectra of Fe₂O₃ NPs/Cu. d) O 1s XPS spectra of Cu*_x_* O/Cu. e) O 1s XPS spectra of Fe₂O₃ NPs/Cu. f) EPR spectra of P‐Cu, Cu*_x_* O/Cu and Fe₂O₃ NPs/Cu.

Plasma treatment is hypothesised to generate a high density of OVs, which can be retained during the formation of Fe₂O₃, benefiting electrochemical performance. To test this expectation, Raman spectroscopy was employed to investigate the defects and local structure of Cu*_x_* O/Cu and Fe₂O₃ NPs/Cu, as shown in Figure 4a. The presence of Cu₂O in Cu*_x_* O/Cu is indicated by strong peaks around 150 and 220 cm⁻¹. Along with the intense 218 cm⁻¹ peak, the peaks around 109 and 298 cm⁻¹ also indicate the presence of CuO.^[^ ^41^ ^]^ The broad peak around 650 cm⁻¹ cannot be attributed to a specific phase as both CuO and Cu₂O exhibit spectral features at this wavenumber.^[^ ^42^ ^]^ The peak intensity suggests that the structure of Cu*_x_* O is not highly ordered or crystalline, likely due to the abundance of lattice defects caused by OVs. For Fe₂O₃ NPs/Cu, peaks at 221, 298, 410, and 610 cm⁻¹ are attributed to Fe₂O₃. The broad shoulder at 610 cm⁻¹ and a weak band at 147 cm⁻¹ are attributed to OVs.^[^ ^43^ ^]^

X‐ray photoelectron spectroscopy (XPS) was used to analyse the surface composition and chemical states of the samples. The Cu Auger LMM spectrum and Cu 2p were used to confirm the valence state of the Cu species (Figures 4b and S5). In P‐Cu and Fe₂O₃ NPs/Cu, Cu is primarily in the zero‐valent state, whereas in Cu*_x_* O/Cu, both monovalent and divalent Cu species coexist.^[^ ^16^ ^]^ It can be inferred that surface Cu is oxidised to Cu*_x_* O during plasma treatment, and Cu*_x_* O begins to decompose into metallic Cu and oxygen at high temperature and in an inert atmosphere. In O 1s XPS spectrum, each O 1s curve were deconvoluted into three components: lattice oxygen (O_l_) at around 529.8 eV, adsorbed oxygen (O_ad_) at around 531.7 eV and adsorbed water (H₂O_ad_) at approximately 533.1 eV (Figure 4d,e).^[^ ^38^ ^]^ The ratio of O_ad_ to O_l_ indicates the content of surface OVs.^[^ ^44^ ^]^ Surface OVs were successfully introduced in Cu*_x_* O/Cu, with a slight increase in content after Fe₂O₃ doping. Figure 4c shows the Fe 2p XPS spectrum, where peaks at 726.1 and 713.1 eV correspond to Fe^3^⁺ 2p₁/₂ and Fe^3^⁺ 2p₃/₂, and peaks at 722.7 and 710.5 eV correspond to Fe^2^⁺ 2p₁/₂ and Fe^2^⁺ 2p₃/₂, respectively.^[^ ^45^, ^46^ ^]^ The Fe^2^⁺ characteristic peaks in the Fe 2p spectrum appear due to electron transfer from VOs after doping. Additionally, the three samples were further analysed using electron paramagnetic resonance (EPR) to quantify unpaired electrons, which proportionally correlate with the amounts of OVs and under‐coordinated dangling bonds. In Figure 4f, the signal intensity at *g* = 2.004 progressively increases with plasma treatment and Fe₂O₃ formation, indicating increased OV content from Cu*_x_* O/Cu to Fe₂O₃ NPs/Cu.^[^ ^47^ ^]^

### Experimental Investigations on NO*_x_* RR Performance of Fe₂O₃ NPs/Cu

**Figure 5:**
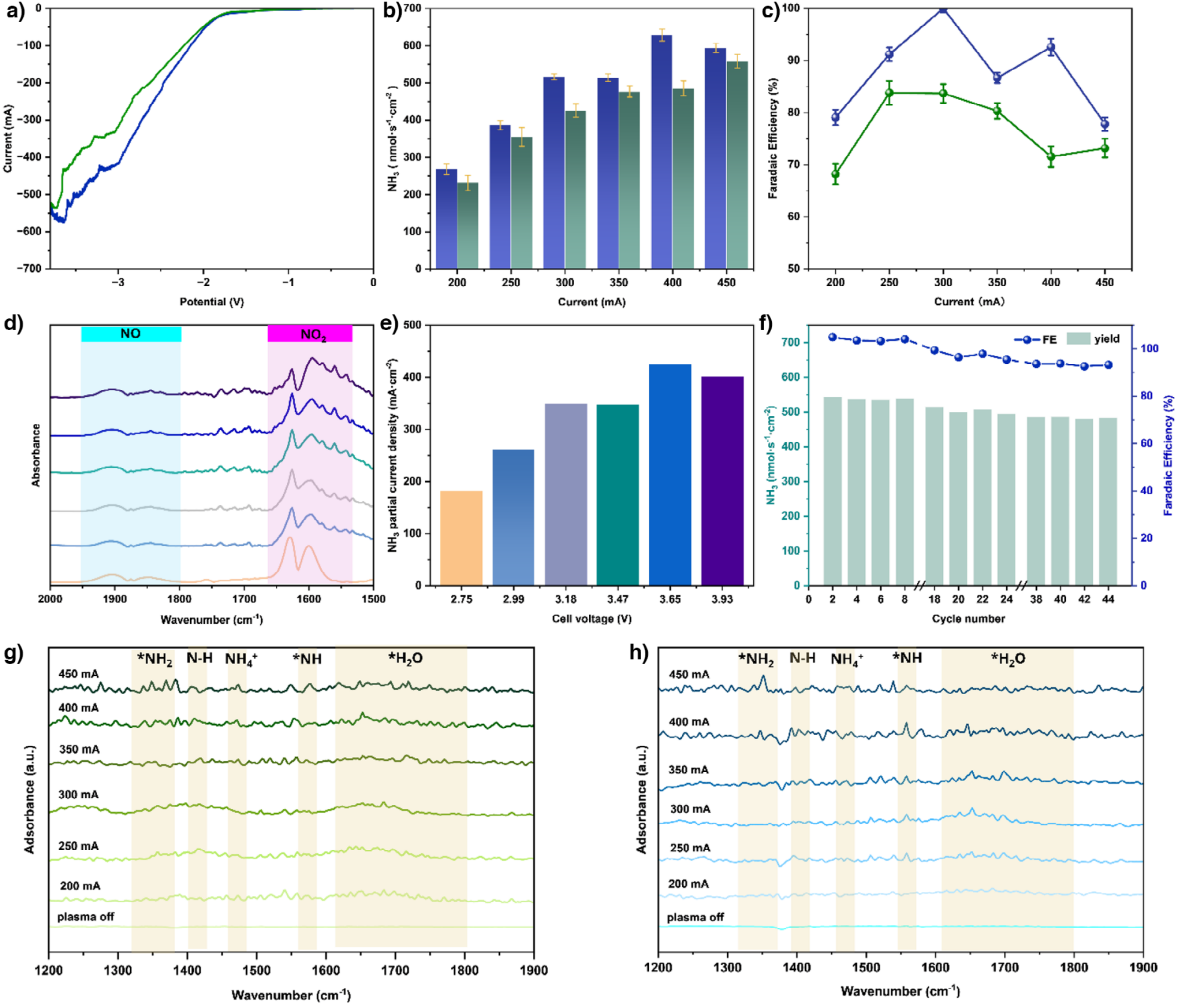
a) LSV curves. b) Ammonia yields at different currents and c) FEs at different currents for Fe₂O₃ NPs/Cu. d) Measured FTIR spectra of reactant and product with Fe₂O₃ NPs/Cu. e) Partial current densities at various cell voltages with Fe₂O₃ NPs/Cu. f) Stability test for Fe₂O₃ NPs/Cu at 300 mA. g) Quasi in situ FTIR spectra of P‐Cu. h) Quasi in situ FTIR spectra of Fe₂O₃ NPs/Cu. Note all current density reported herein is normalised to the geometric surface area without any IR compensation.

To verify the effectiveness of the GP‐CE system, Fe₂O₃ NPs/Cu was used as a cathode and tested under ambient conditions. The linear sweep voltammetry (LSV) curves presented in Figure S6a show a more intense current for the plasma‐on condition than the plasma‐off condition. Gas chromatography (GC) was used to analyse the reaction product when the plasma was off (Figure S6b), showing a clear tendency towards HER without plasma activation. The fact that NH₃ was only produced when air was activated by plasma demonstrates the effectiveness of the reaction pathway. Subsequently, the impact of Fe₂O₃ doping was investigated. The current density significantly increased after doping, demonstrating that the successful introduction of Fe₂O₃ NPs on Cu effectively enhances electrocatalytic activity (Figure 5a). The reactant was confirmed by infrared absorption spectroscopy (FTIR) before and after each reaction (Figure S7). The absolute concentrations of N₂O, NO and NH₃ were determined by fitting the FTIR spectra to simulated results based on parameters from the HITRAN database.^[^ ^48^ ^]^ The species concentration was adjusted to best align with the experimentally measured spectra.^[^ ^49^ ^]^ As shown in Figure 5d, NO and NO₂ were rapidly consumed, whereas NH₃ was generated. During the batch experiment, the total concentration of nitrogen‐containing species remained constant as the inflow rate remained steady. The produced NH₃ was determined spectrophotometrically using Nessler's method,^[^ ^50^ ^]^ and the corresponding calibration is provided in Figure S8. Based on chronogalvanostatic curves (Figure S9) and UV–vis absorption spectra (Figure S10), NH₃ yield and FEs were obtained at current densities ranging from −200 to −450 mA. Cell voltage variations at each current show that Fe₂O₃ NPs/Cu outperforms its counterpart in both energy efficiency and catalytic activity. As shown in Figure 5b,c, Fe₂O₃ NPs/Cu achieves an NH₃ yield of 628 nmol·s⁻¹·cm⁻^2^ at 400 mA and an FE of 100% at 300 mA, both higher than those obtained by P‐Cu.

Moreover, Figure S11 shows that Cu*_x_* O/Cu delivers inferior performance compared to P‐Cu, indicating that the superior electrocatalytic NRR performance mainly originates from Fe₂O₃ doping. As shown in Figure 5e, NH₃ partial current density reaches a maximum value and then decreases with further increases in cell voltage due to the increasing HER. The cell voltage generally remains below 4 V in all tests with the Fe₂O₃ NPs/Cu catalyst. Quasi in situ FTIR was carried out to observe the dynamic interactions between catalysts surface and intermediates during the reaction process. Figure 5g,h illustrates that as current increases, the Fe₂O₃ NPs/Cu catalyst shows stronger absorption bands of *NH_2_ (1300–1360 cm^−1^), N─H (≈1400 cm^−1^), *NH (≈1560 cm^−1^) than that of P‐Cu as well as the generated NH_3_ (NH_4_^+^, ≈1470 cm^−1^), reflecting the superior hydrogenation ability of Fe₂O₃ NPs/Cu.^[^ ^51^, ^52^ ^]^ Electrocatalytic stability was evaluated by collecting the product every 0.5 h for a total of 44 cycles. As shown in Figure 5f, no significant change in NH_3_ production and FE are observed during the first 8 cycles and the yield and FE have slightly decreased but remain stable at a certain level (yield ∼500 nmol·s^−1^·cm^−2^, FE ∼90%) as a function of time. The chronogalvanostatic curve in Figure S12 obtained during the stability test shows minor voltage fluctuations due to electrolyte renewal. After testing, Fe₂O₃ NPs/Cu were subjected to SEM and HRTEM characterisation, and Figure S13 shows that the catalyst retained its morphology with no in situ reduction of Fe₂O₃. Figures S14 and S15 present the XRD and XPS spectra, respectively, revealing no changes in crystal structure or valence state, which demonstrate good stability of Fe₂O₃ NPs/Cu. As summarised in Table S1, these results confirm that highly defective Fe₂O₃ NPs/Cu effectively enhance both activity and selectivity. These findings experimentally affirm the role of Fe₂O₃ NPs/Cu in improving electrochemical performance.

### Theoretical Investigations on NO*_x_* RR Performance of Fe₂O₃ NPs/Cu

**Figure 6:**
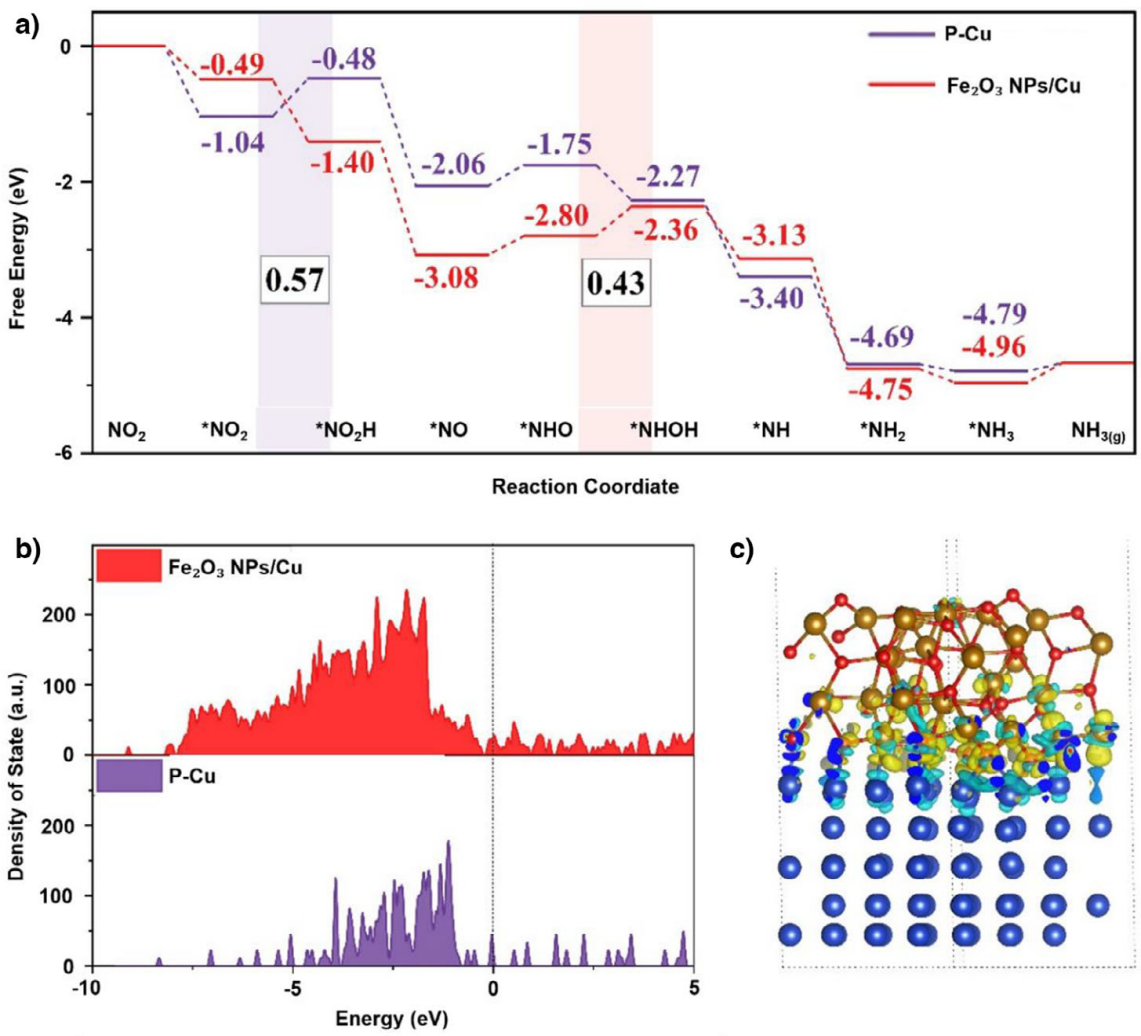
a) Gibbs free energy diagram of the NO*_x_* RR on the Fe₂O₃ NPs/Cu and Cu‐110 surfaces of P–Cu. b) Calculated projected density of states (PDOS) analysis of the Fe 3d orbitals of Fe_2_O_3_ NPs/Cu and the Cu 3d orbitals of P‐Cu. c) Differential charge density at the Fe_2_O_3_ NPs/Cu interface, where cyan and yellow represent charge depletion and accumulation, respectively.

To further understand the mechanism of NO*_x_* reduction to NH_3_ and the differences in activity between Fe₂O₃ NPs/Cu and P‐Cu, we conducted DFT simulations. Based on experimental and characterisation results, we constructed models for Fe₂O₃ NPs/Cu and Cu‐110 of P‐Cu, as shown in Figure S16. We investigated two typical reaction pathways for each system: the NOH pathway (*NO → *NOH → *N → *NH) and the NHO pathway (*NO → *NHO → *NHOH → *NH). Detailed reaction free energy diagrams and corresponding structures for each step are provided in Figures S17–S19. Total energy of absorbate‐slab system and free energy correction for reaction intermediates in Gibbs free energy calculation are provided in Table S2. Figure 6a illustrates the optimal reaction pathways for both catalysts, which are found to be the NHO pathway. For P‐Cu, the rate‐determining step (RDS) is *NO_2_ → *NO_2_H with an energy barrier of 0.54 eV. In contrast, Fe₂O₃ NPs/Cu facilitates the hydrogenation of *NO_2_, making this step thermodynamically favourable. The RDS for Fe₂O₃ NPs/Cu is *NHO → *NHOH with an energy barrier of 0.43 eV, which is lower than that of Cu‐110, thus exhibiting superior performance. To further understand the reaction mechanism, we studied the electronic structures of Fe₂O₃ NPs/Cu and P‐Cu. Compared to P‐Cu, Fe₂O₃ NPs/Cu exhibits a more continuous and abundant density of states (DOS) near the Fermi level, indicating enhanced electrical conductivity and promoting electron transfer at the heterostructure interface (Figure 6b).^[^ ^53^ ^]^ Additionally, the differential charge density reveals significant charge accumulation at the Fe₂O₃ NPs/Cu interface, confirming strong charge transfer capability (Figure 6c). The calculation results indicate that Fe₂O₃ NPs/Cu is more liable for NO*_x_* electrocatalytic reduction reaction.

## Conclusion

In summary, we report an Fe₂O₃ NPs/Cu catalyst for ammonia production. Abundant defects are induced using a multifunctional oxygen vacancies strategy from plasma pretreatment combined with wet chemical calcination. This method makes it possible to control the modulation of oxygen vacancies during fabrication by the bombardment of high‐energy oxygen species and redistribution of the local electronic structure. Benefiting from the superior electrical conductivity and promoting electron transfer at the heterostructure interface, the Fe₂O₃ NPs/Cu catalyst achieves an excellent ammonia production rate of 628 nmol·s⁻¹·cm⁻^2^, along with nearly 100% faradaic efficiency at 300 mA, which outperforms the Cu‐based catalysts for NO reduction reactions reported to date. In this regard, our study presents a successful attempt to design efficient electrocatalysts for an effective air‐to‐NH₃ process.

## Conflict of Interests

Authors Cullen and Zhang are associated with Plasmaleap Technologies, a commercial entity, with interest in plasma‐driven ammonia synthesis.

## Supporting information



Supporting Information

## Data Availability

The data that support the findings of this study are available from the corresponding author upon reasonable request.
