# Supplementary material for: Regulating Multifunctional Oxygen Vacancies for Plasma‐Driven Air‐to‐Ammonia Conversion
Source: Angew Chem Int Ed Engl. 2025 May 12;64(27):e202508240. doi: 10.1002/anie.202508240 (PMC12207347; doi:10.1002/anie.202508240)
Supplement: Supplementary file 1 — Supporting Information [file ANIE-64-e202508240-s001.docx]

Supporting Information

**Regulating Multifunctional Oxygen Vacancies for Plasma-Driven Air-to-Ammonia Conversion**

Wanping Xu, Jiaqian Wang, Tianqi Zhang, Jungmi Hong, Qiang Song, Zhongkang Han*, Patrick Cullen*

Table of Contents

[Experimental Section	3](#_Toc195137395)

[Chemicals	3](#_Toc195137396)

[Preparation of catalysts	3](#_Toc195137397)

[Characterization	3](#_Toc195137398)

[Electrocatalytic Measurements	4](#_Toc195137399)

[DFT calculations	5](#_Toc195137400)

[Supporting Figures	6](#_Toc195137401)

[Figure S1.	6](#_Toc195137402)

[Figure S2.	7](#_Toc195137403)

[Figure S3.	8](#_Toc195137404)

[Figure S4.	9](#_Toc195137405)

[Figure S5.	10](#_Toc195137406)

[Figure S6.	11](#_Toc195137406)

[Figure S7.	12](#_Toc195137407)

[Figure S8.	13](#_Toc195137408)

[Figure S9.	14](#_Toc195137409)

[Figure S10.	15](#_Toc195137410)

[Figure S11.	16](#_Toc195137411)

[Figure S12.	17](#_Toc195137412)

[Figure S13.	18](#_Toc195137413)

[Figure S14	19](#_Toc195137413)

[Figure S15.	20](#_Toc195137413)

[Figure S16.	21](#_Toc195137414)

[Figure S17.	22](#_Toc195137415)

[Figure S18.	23](#_Toc195137416)

[Figure S19.	24](#_Toc195137417)

[Supporting Table	25](#_Toc195137418)

[Table S1.	25](#_Toc195137419)

[Table S2.	26](#_Toc195137419)

[Supporting References	27](#_Toc195137420)

[Author Contributions	28](#_Toc195137421)

# Experimental Section

# Chemicals

All chemicals were purchased from Sigma-Aldrich and used without further purification. Milli-Q water with a resistivity of 18.2 MΩ·cm was obtained from a Purelab Quest water purification system. The proton exchange membrane (Nafion 117) used in this work was purchased from SCI Materials Hub. Compressed air (instrument grade), ultra-high purity O₂ (99.999%), and N₂ (99.999%) were purchased from BOC Gas, Australia.

# Preparation of catalysts

The commercial 8000-mesh Cu was cut into 1 × 1 cm² pieces. The Cu mesh was sequentially immersed in acetone, 0.1 M H₂SO₄, and Milli-Q water for 30 minutes each to remove organic impurities and oxide layers. The cleaned Cu mesh was then dried in a vacuum oven at 60 °C to obtain P-Cu. As shown in Figure S1a, P-Cu was placed in a dielectric barrier discharge (DBD) quartz chamber reactor with a 10 kHz AC plasma power supply (100 W) and treated for 1 minute on each side with 200 sccm of O₂ to obtain Cu_x_O/Cu. Next, 0.5 g of FeCl₃·6H₂O was dissolved in 50 mL of DI water, and Cu_x_O/Cu was dispersed in the solution. The mixed solution was stirred and left to stand for 4 hours, followed by drying in a vacuum oven at 60 °C for 1 hour. The dried sample was transferred to a tube furnace and annealed at 800 °C for 1 hour at a heating rate of 5 °C/min in an N₂ atmosphere to produce Fe₂O₃ NPs/Cu.

# Characterization

XRD measurements were conducted on a Rigaku Smartlab SE diffractometer using Cu Kα radiation (40 kV, 4 mA, λ = 0.15406 nm). SEM and EDS mapping were performed on a Zeiss Sigma VP HD. TEM and STEM analyses were carried out using a JEOL JEM-2200FS. For TEM analysis, the samples were prepared by ultrasonic dispersal in ethanol, and the resulting suspension was deposited onto a holey carbon film-coated copper microgrid. XPS measurements were performed using a Thermo Fisher ESCALAB 250Xi spectrometer with a monochromatic Al Kα source (1486.6 eV). Peak fitting of the high-resolution data was carried out using Thermo Avantage V 6.1 surface chemical analysis software, with all spectra calibrated by aligning the adventitious carbon C 1s core level to 284.8 eV. Raman spectroscopy was performed using a Renishaw inVia Qontor Raman spectrometer with photoluminescence capabilities. UV-Vis measurements were performed using a Shimadzu UV-3600 UV-VIS-NIR spectrophotometer. FTIR spectroscopy was conducted using a Bruker INVENIO-R FT-IR spectrometer with gas cell (A131-0100/TR) in the wavenumber range of 4000 to 800 cm⁻¹ at a resolution of 4 cm⁻¹. Simulated standard spectra were generated with a wavenumber computational step of 1 cm⁻¹, an optical path of 10 cm, an assumed Lorentzian function, and an apparatus resolution of 10 cm⁻¹. GC analysis was performed using a Shimadzu Nexis GC-2030.

# Electrocatalytic Measurements

A Suman CTP-2000K power supply was used as the plasma generator, and all electrochemical measurements were carried out using a Metrohm Autolab M204 multichannel and VIONIC electrochemical workstation. In a typical experiment, a constant air flow of 100 sccm was introduced into a quartz tubular plasma spark discharge reactor (6 mm ID, 10 mm OD), equipped with two flat-end stainless-steel electrodes (3.175 mm OD, 10 mm discharge gap), and powered by a 10 kHz sinusoidal power supply at 15 W. The outlet gas was directed into a custom-designed membrane electrode assembly (MEA), where the prepared cathode and commercial Ni foam anode were separated by a Nafion 117 membrane, and 1M KOH was circulated as the anolyte through the anode at a flow rate of 50 mL/min. The Nafion 117 membrane was pretreated by immersion in 5% H₂O₂ (60 min), H₂O (60 min), 0.5 M H₂SO₄ (60 min), and H₂O (60 min) at 80°C. The total amount of NH₃ produced was determined by the colorimetric method. A 50 µL solution containing NH₃ was diluted with DI water to 1 mL, and 40 µL of Nessler’s reagent was added to the solution. After 10 minutes, the solution was analysed for UV–vis absorbance measurement at 420 nm, and the NH₃ concentration was calculated based on the light absorbance.

**Calculation of NH_3_ yield and FE**

**NH_3_ yield could be calculated using the following formula**

$${NH}_{3} yield=\frac{n\left( {NH}_{3} \right)}{A\times t}$$

where n is the amount t of produced NH_3_, A is effective area of electrode, t is reaction time.

**FE could be calculated using the following formula**

For NO to NH_3_

$$FE=\frac{5\times F\times n({NH}_{3})}{Q}$$

For NO_2_ to NH_3_

$$FE=\frac{7\times F\times n({NH}_{3})}{Q}$$

Where F is Faraday constant (96485 C mol^–1^), Q is total charge consumption. The reactants involved in the reaction and the amount of electric charge were determined by FTIR tests before and after the electrochemical.

# DFT calculations

In this study, we performed first-principles computations using the Vienna Ab-initio Simulation Package (VASP) based on density functional theory (DFT).^1^ The Perdew-Burke-Ernzerhof (PBE) ^2^ generalized gradient approximation (GGA) ^3^was employed to describe exchange and correlation effects. Structural optimization was carried out using the Gamma-centered Monkhorst-Pack grids with a 2 × 2 × 1 K-point mesh and an energy cutoff of 520 eV. To prevent interactions between periodic layers, a vacuum layer of 15 Å was introduced along the z-direction.

**The adsorption energy was calculated using the following formula:**

$$E_{ads}=E_{x/slab}-\left[ E_{slab}+E_{x} \right]$$

Here, E_x/slab_ represents the complete energy of the slab with the adsorbed species at equilibrium, E_slab_ stands for the energy of the unadorned slab, and E_x_ refers to the energy of the isolated adsorbate in its gaseous form.

**The variation in Gibbs free energy (ΔG) was ascertained through:**

$$\Delta G=E_{ads}+E_{ZPE}-TS$$

In this equation, E_ZPE_ is the zero-point energy determined via VASP, while TS signifies the entropy contribution at 298.15 K. Note that E_ZPE_ and S can be directly obtained from frequency calculations using VASPKIT.^4^

# Supporting Figures

Figure S1. (a) Plasma reactor for P-Cu treatment, (b) Scheme for GP-PE system. Gas product (NO_x_) was quantified by FTIR.

Figure S2. (a) SEM image of P-Cu, (b) SEM image of Cu_x_O/Cu.

Figure S3. EDS mapping images of (a) P-Cu, (b) Cu_x_O/Cu, (c) Fe_2_O_3_ NPs/Cu.

Figure S4. XRD patterns of P-Cu, Cu_x_O/Cu, Fe_2_O_3_ NPs/Cu.

Figure S5. Cu 2p XPS spectra of (a) P-Cu, (b) Cu_x_O/Cu, (c) Fe_2_O_3_ NPs/Cu.

Figure S6. (a) LSV curves of Fe_2_O_3_ NPs/Cu under plasma on and plasma off conditions, (b) Faradaic efficiency for H_2_ production under plasma off conditions with Fe_2_O_3_ NPs/Cu.

Figure S7. (a) The original FTIR data of the gases before and after the reaction under plasma on conditions. (b) The gas content ratios after simulation correction.

Figure S8. UV-vis absorption spectra of NH_3_ at various concentrations after being incubated and the corresponding calibration curve.

Figure S9. Chronogalvanostatic curves at different currents of (a) P-Cu, (b) Cu_x_O/Cu, (c) Fe_2_O_3_ NPs/Cu, (d) integrated average values.

Figure S10. UV-vis absorption spectrum after a 20-fold dilution of (a) P-Cu, (b) Cu_x_O/Cu, (c) Fe_2_O_3_ NPs/Cu at each current for 10 min.

Figure S11. Comparison of the (a) NH_3_ yield, (b) FEs for P-Cu and Cu_x_O/Cu.

Figure S12. Long-term stability test**.**

Figure S13. (a) SEM image and (b) HRTEM image of Fe_2_O_3_ NPs/Cu nanofiber after the durability test.

**Figure S14.** XRD pattern of Fe_2_O_3_ NPs/Cu after the durability test.

**Figure S15.** (a) O 1s XPS spectra, (b) Fe 2p XPS spectra, (c) Cu 2p XPS spectra, (d) XPS survey spectra of Fe₂O₃ NPs/Cu after the durability test.

Figure S16. Optimized structural models of (a) P-Cu and (b) Fe_2_O_3_ NPs/Cu. The blue, gold and red spheres represent Cu, Fe, and O atoms, respectively.

Figure S17. Free energy diagrams of two pathways for the reduction of NO_x_ to NH_3_ on (a) P-Cu and (b) Fe_2_O_3_ NPs/Cu.

Figure S18. optimized structures of the intermediates for NO_x_ to NH_3_ on P-Cu. The blue, grey, pink and red spheres represent Cu, N, H, and O atoms, respectively.

Figure S19. optimized structures of the intermediates for NO_x_ to NH_3_ on Fe_2_O_3_ NPs/Cu. The blue, gold, grey, pink, and red spheres represent Cu, Fe, N, H, and O atoms, respectively.

# Supporting Table

Reported Electrochemical reduction of NO_x_ to NH_3_ usually occurs in aqueous electrolyte solution, in which the NO molecule directly adsorbs on the active sites and is reduced into NH_3_, whereas the NO_2_ molecule easily reacts with water to produce NO_3_^−^ and NO. Therefore, NO gas is chosen as representative nitrogenous sources for the NO_x_ reduction reaction for electrosynthesis of ammonia to compare the activity of our catalyst. Additionally, a comparison of the catalysts used in the plasma-assisted conversion of NOₓ followed by electrochemical reduction to NH₃ is also presented.

Table S1. NO_x_RR performance comparison between recently reported Cu-based catalysts.

The Gibbs free energy changes (ΔG) were calculated through:

ΔG = E_ads_ + ΔE_ZPE_ + ΔE_H_ – TΔS = E_ads_ + ΔG_correct_

In this equation, where ΔE_ZPE_ and ΔE_H_ represent the zero-point energy and thermal enthalpy, respectively, and TΔS denotes the entropy contribution at 298.15 K. The free energy correction (ΔG_correct_) can be directly obtained from the vibrational frequency calculations through the VASPKIT.^4^

Table S2. **Total energy of adsorbate-slab systems (E_x/slab_) and free energy correction (ΔG_correct_) for reaction intermediates in Gibbs free energy calculations for Fe₂O₃ NPs/Cu and P-Cu surfaces.**

**Sup****porting** **References**

[1] G. Kresse, J. F. l., Phys. Rev. B 1996, 54, 11169.

[2] John P. Perdew, K. B., Yue Wang, Phys. Rev. B 1996, 54, 16533.

[3] John P. Perdew, K. B., Matthias Ernzerhof, Phys. Rev. Lett. 1996, 77, 3865.

[4] Wang, V., Xu, N., Liu, J.-C., Tang, G.,Geng, W.-T., Comput. Phys. Commun. 2021, 267, 108033.

[5] Chen, K., Xiang, J., Guo, Y., Liu, X., Li, X.,Chu, K., Nano Lett. 2024, 24, 541-548.

[6] Ko, B. H., Hasa, B., Shin, H., Zhao, Y.,Jiao, F., J. Am. Chem. Soc. 2022, 144, 1258-1266.

[7] Wang, D., Zhu, X., Tu, X., Zhang, X., Chen, C., Wei, X., Li, Y.,Wang, S., Adv. Mater. 2023, 35, e2304646.

[8] Wu, Z., Liu, Y., Wang, D., Zhang, Y., Gu, K., He, Z., Liu, L., Liu, H., Fan, J., Chen, C.,Wang, S., Adv. Mater. 2024, 36, e2309470.

[9] Shi, J., Wang, C., Yang, R., Chen, F., Meng, N., Yu, Y.,Zhang, B., Sci. China Chem. 2021, 64, 1493-1497.

[10] Krzywda, P. M., Paradelo Rodríguez, A., Benes, N. E., Mei, B. T.,Mul, G., ChemElectroChem 2022, 9, e202101273.

[11] Wei L., Mengyang X., Chao Z., Ben C., Jiahe C., He L., Honghui O., Guidong Y., *Nat. Commun.* 2024,15, 3524.

[12] Yongwen R., Chang Y., Linshan W., Xinyi T., Zhao W., Qianbing W., Yafang Z., Jieshan Q., *J. Am. Chem. Soc*. 2022, 144, 10193.

[13] Jing S., David A., Rahman D., Hassan M., Tianqi Z., Renwu Z., Patrick J. C., Emma C. L., Ali (Rouhollah) J., Rose A., *Energy Environ. Sci.*, 2021,14, 865.

[14] Wenyi L., Shengbo Z., Jun D., Jiafang L., Zhiwei W., Haimin Z., Jun D., Longwei C., Changhao L., *Sustain. Chem. Eng.*, 2023, 11, 1168.

# Author Contributions

W.X. conceived the idea, synthesized the materials, performed characterizations and electrochemical experiments, analysed the data, and wrote the manuscript. J.W. conducted the density functional theory simulations. T.Z. designed the reaction system and performed the FTIR experiments. J.H. carried out the FTIR simulations. Q.S. participated in data analysis. Z.H. supervised the multiphysics simulations. P.J. supervised the project. All authors participated in the discussion and editing of the manuscript.
